# Enhanced anti-metastatic bioactivity of an IGF-TRAP re-engineered to improve physicochemical properties

**DOI:** 10.1038/s41598-018-35407-2

**Published:** 2018-11-26

**Authors:** George Vaniotis, Serge Moffett, Traian Sulea, Ni Wang, S. Mehdy Elahi, Etienne Lessard, Jason Baardsnes, Stephanie Perrino, Yves Durocher, Jan Frystyk, Bernard Massie, Pnina Brodt

**Affiliations:** 1 Department of Surgery, McGill University, Montreal Quebec, Canada; 2 Department of Medicine, McGill University, Montreal Quebec, Canada; 3 Department of Oncology, McGill University, Montreal Quebec, Canada; 4 Institute of Parasitology, McGill University, Montreal Quebec, Canada; 5 Cancer Research Program, Research Institute of the McGill University Health Center, Montreal Quebec, Canada; 6 Human Health Therapeutics Research Centre, National Research Council Canada, Montreal Quebec, Canada; 7 Department of Clinical Medicine, Aarhus University, Aarhus, Denmark

## Abstract

The insulin-like growth factor (IGF) axis has been implicated in the progression of malignant disease and identified as a clinically important therapeutic target. Several IGF-1 receptor (IGF-1R) targeting drugs including humanized monoclonal antibodies have advanced to phase II/III clinical trials, but to date, have not progressed to clinical use, due, at least in part, to interference with insulin receptor signalling. We previously reported on the production of a soluble fusion protein consisting of the extracellular domain of human IGF-1R fused to the Fc portion of human IgG_1_ (first generation IGF-TRAP) that bound human IGF-1 and IGF-2 with a 3 log higher affinity than insulin. We showed that the IGF-TRAP had potent anti-cancer activity in several pre-clinical models of aggressive carcinomas. Here we report on the re-engineering of the IGF-TRAP with the aim of improving physicochemical properties and suitability for clinical applications. We show that cysteine-serine substitutions in the Fc hinge region of IGF-TRAP eliminated high-molecular-weight oligomerized species, while a further addition of a flexible linker, not only improved the pharmacokinetic profile, but also enhanced the therapeutic profile of the IGF-TRAP, as evaluated in an experimental colon carcinoma metastasis model. Dose-response profiles of the modified IGF-TRAPs correlated with their bio-availability profiles, as measured by the IGF kinase-receptor-activation (KIRA) assay, providing a novel, surrogate biomarker for drug efficacy. This study provides a compelling example of structure-based re-engineering of Fc-fusion-based biologics for better manufacturability that also significantly improved pharmacological parameters. It identifies the re-engineered IGF-TRAP as a potent anti-cancer therapeutic.

## Introduction

The insulin-like growth factor (IGF) axis consisting of the heterotetrameric IGF-1 receptor (IGF-1R) and its high affinity binding ligands IGF-1 and IGF-2 have been implicated in all stages of cancer growth and progression including cellular transformation, epithelial-to-mesenchymal transition (EMT), invasion and metastasis, as well as in the regulation of the tumor microenvironment^1–5^. The evidence that the IGF-axis plays a critical role in malignancy has led to an intensive effort to develop anti-cancer drugs that target the IGF-1R. Two main strategies have been used to date, namely, monoclonal anti-IGF-1R antibodies that block ligand binding, and small-molecule tyrosine kinase inhibitors (TKIs) that block receptor signalling. Eight IGF-1R targeting monoclonal antibodies and 6 TKIs have been evaluated clinically, as either monotherapy or in combination with chemotherapy or other targeted therapies^6–8^. However, the results were generally disappointing as little or no benefit was documented in phase II/III clinical trials. Several factors have been proposed as contributing to this lack of efficacy, namely: (i) compensatory growth signaling by insulin receptor (IR) isoform A that binds IGF-2 with high affinity and is highly expressed on some cancer cells; (ii) signaling by hybrid receptor IR:IGF-1R that can bind insulin and IGF; (iii) compensatory signaling and ERK activation by other receptor tyrosine kinases (RTKs); (iv) the potential role of nuclear IGF-1R in promoting tumor cell proliferation^9^; and (v) the lack of biomarkers that could aid in identifying a responsive patient population. In addition to lack of efficacy, the high homology between IR and IGF-1R has resulted in undesirable side effects, including hyperinsulinemia and hyperglycemia following treatment with some of these inhibitors.

These obstacles to therapeutic efficacy have led to development of a new therapeutic strategy with a class of drugs that target the ligands instead of the receptor. An antibody that binds IGF-1 and IGF-2 with high affinity was recently developed by MedImmune (MEDI-573) and is currently in a phase II clinical trial^10^. Another ligand neutralizing antibody, MAb BI 836845 (Xentuzumab) developed by Boehringer Ingelheim, has advanced to a phase I clinical trial (ClinicalTrials.gov; NCT01403974) and is now in phase Ib, in combination with a CDK4/6 inhibitor for HR^+^/HER2^−^ metastatic breast cancer and other solid tumors^11^.

Another effective strategy for blocking the action of cell surface receptors is the use of soluble receptor decoys that bind the ligands with high affinity, thus reducing their bioavailability to the cognate receptor in a highly specific manner^12–14^. For example, a soluble TNF-α receptor-Fc fusion protein (TNF-TRAP, Etanercept or Enbrel) is currently in routine clinical use for the treatment of inflammatory conditions such as rheumatoid arthritis^13^ and a VEGFR1/VEGFR2-Fc decoy (VEGF-TRAP, Aflibercept) is approved for the treatment of wet macular degeneration under the trade name Eylea, and for metastatic colorectal cancer as Zal-TRAP^12^.

We previously reported on the production of a soluble fusion protein consisting of the extracellular domain of human IGF-1R fused to the Fc portion of human IgG_1_ (the IGF-TRAP)^15^. We showed that the IGF-TRAP bound human IGF-1 and IGF-2 with high affinities and human insulin with a 1000-fold lower affinity, mimicking the K_D_ values reported for the native receptor. Moreover, we showed that the IGF-TRAP had significant anti-neoplastic activity in several highly aggressive pre-clinical carcinoma murine models, including against triple negative breast cancer, colorectal and lung carcinoma cells^15^.

One problem frequently encountered with Fc-fusion proteins is the formation of high-molecular-weight (HMW) complexes due to irrelevant disulfide bonding between adjacent Fc fragments^16^. Indeed, we observed HMW species that migrated at a mass corresponding to oligomerized molecules in polyacrylamide gels electrophoresis (PAGE) of IGF-TRAP preparations^15^. We therefore re-engineered the IGF-TRAP to eliminate such aberrant disulfide bonding by cysteine-to-serine substitutions in the hinge region of the human IgG_1_ Fc fragment of the IGF-TRAP, as well as incorporation of a longer and more flexible linker between the IGF-1R ectodomain and the Fc domain. Here we describe the properties of the modified IGF-TRAPs, their improved biophysical properties and their unexpectedly distinct biological and better therapeutic activities.

## Materials and Methods

### Cells

All procedures and protocols described in this manuscript were approved by the biohazard committees of the respective institutions. The origins and metastatic properties of murine colon carcinoma MC-38 and their culture conditions and testing have been described in detail elsewhere^17^. MC-38 cells were originally from an NCI repository and were obtained as a kind gift from Dr. Shoshana Yakar (New York University, NY). They were recently authenticated by Didion and colleagues^18^ using SNP profiling, as described. Human embryonic kidney 293 (HEK293) cells transfected with the human IGF-1R gene were used for the kinase receptor activation (KIRA) assay and have been previously described^19^.

### Mice

All mouse experiments were carried out in strict accordance with the recommendations of the Canadian Council on Animal Care (CCAC) “Guide to the Care and Use of Experimental Animals” and under the conditions and procedures approved by the Animal Care Committee of McGill University (AUP # 5733). C57Bl/6 female mice were bred in the animal facility of the Research Institute of the McGill University Health Center. A colony of mice with an inducible, liver specific IGF-I deficiency (iLID) was maintained in the same facility and their phenotype confirmed as described in detail previously^3^. In these mice, a single tamoxifen injection results in gene recombination by activating the Cre recombinase gene under the control of a liver specific anti-trypsin 1α promoter. The inducible *igf1* deletion results in 50–70% reductions in serum IGF-I levels within ~18 hr of a single i.p. injection of 0.3 mg tamoxifen per mouse^3,20^. The colony was maintained as per the guidelines of the McGill University Animal Care Committee, newborn mice routinely genotyped and reduced IGF-I plasma levels in the mice confirmed by ELISA^20^.

### Reagents

Recombinant human (rh) IGF-1 for KIRA assay was obtained from the National Institute of Biological Standards and Calibrators (NIBSC, Hertfordshire, UK) (02/254). rhIGF-1 and rhIGF-2 and insulin used for surface plasmon resonance (SPR) experiments were from Sigma-Aldrich (Oakville, ON, Canada) and recombinant mouse IGF-1 was from Cedarlane (Burlington, ON, Canada). The phospho-IGF-1R ELISA kit was from R&D Systems (Minneapolis, MN). Indium^111^ was from MDS Nordion Inc. (Ottawa, ON, Canada).

### Structural optimization of the IGF-TRAP

The three-dimensional (3D) structure of the (αβ)_2_ heterotetrameric human IGF-1R ectodomain was inferred from the crystallographic structure of human IR (PDB codes 3LOH and 4ZXB) and its recent structural refinements^21,22^, based on their similar multidomain organization, sequence identity (57%), and shared ligands. The 3D structure of the human IgG_1_ Fc fragment, including the hinge region was based on the crystal structure of a full-length antibody (PDB code 1HZH)^23^, whereas its binding mode to human FcRn was based on the crystal structure of the Fc-FcRn-albumin complex^24^. The required lengths for flexible linkers were calculated from the structure-based linear distance for linkage (Å) divided by a factor of 2.5 based on the Cα-Cα extent of fully extended linkers, which peaks at 3.0 Å^25^, minus an average tolerance of 0.5 Å per amino-acid residue to allow for deviations of the linker path from linearity (see Supplementary Fig. S1). Structural visualization, manipulations and measurements were performed in the PyMOL software (Schroedinger, Inc., NY, NY).

### Generation of modified IGF-TRAPs

**Figure 1:**
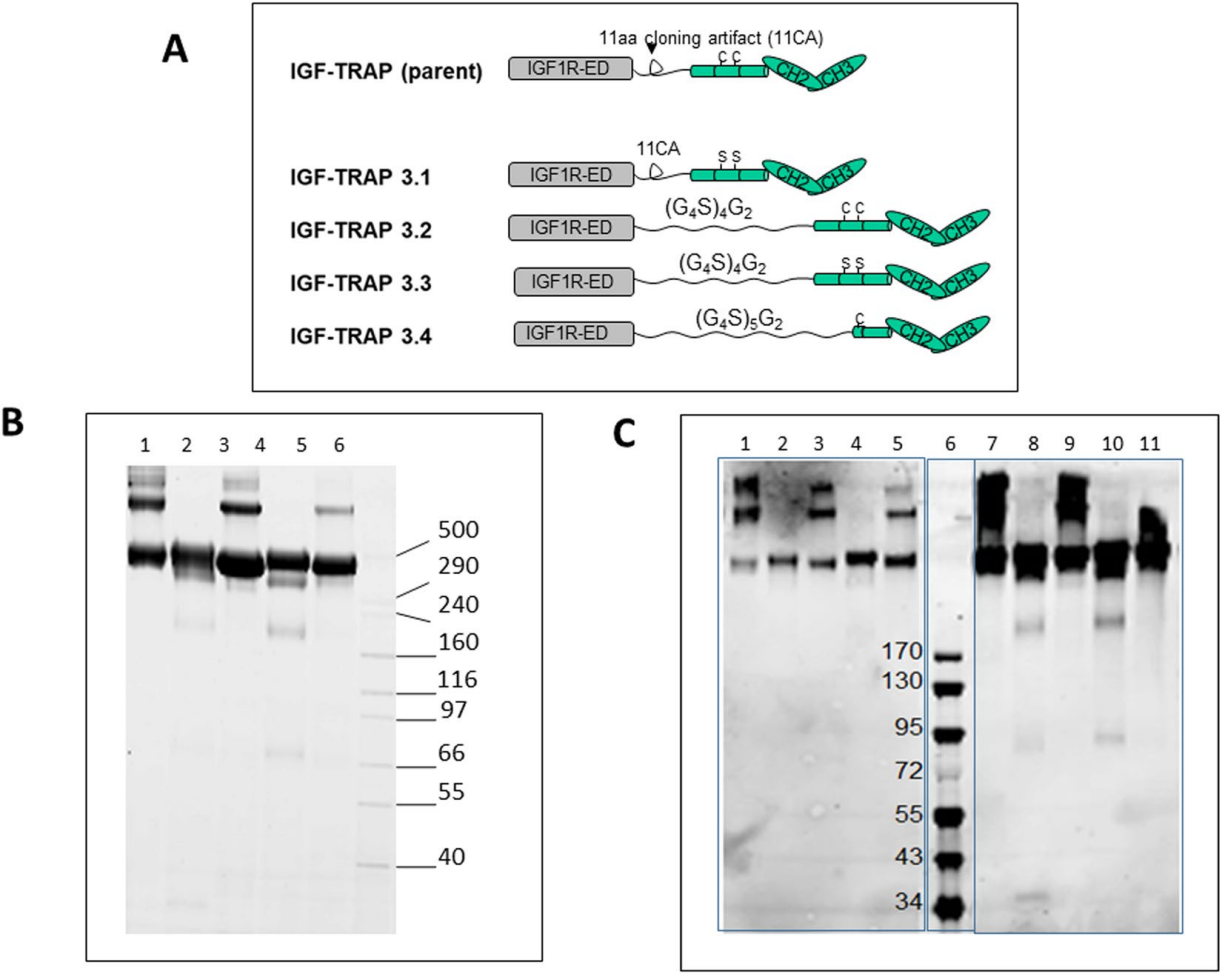
Cysteine-serine substitutions in the Fc domain of the IGF-TRAP reduce HMW oligomers. Shown in (**A**) is a schematic representation of the modifications engineered in the parent IGF-TRAP and in (**B**) results of SDS-PAGE performed on purified parental or modified IGF1R-hFc-IgG_1_ proteins (5 μg loaded per lane) using denaturing and non-reducing condition. Shown are in lane 1 – parent IGF-TRAP; lane 2 – IGF-TRAP 3.1; lane 3 – IGF-TRAP 3.2; lane 4 – IGF-TRAP 3.3; lane 5 – IGF-TRAP 3.4; lane 6 – HMW protein standard (Invitrogen). Shown in (**C**) are Western blots performed with an antibody to IGF1R-α (SC-7952, lanes 1–5) and with anti-Fc antibodies (Cy5-goat-anti-human IgG, lanes 7–11). Lanes 1 & 7 are results with the parental IGF-TRAP and subsequent lanes are ordered as above for (B). Lane 6 – EZ-Run pre-stained Protein Ladder (Fisher).

The sIGF1R-hFc-IgG1 (parent Trap) was originally cloned into the BamHI site of a SmaI-NdeI deleted pUC19 plasmid. This modified pUC19 plasmid was used as a backbone for further subcloning and structural modifications. The modified sequences were synthesized by Genescript and subcloned into the pUC57 vector. Four modified structures designated IGF-TRAP-3.1, -3.2, -3.3 and -3.4 were generated as detailed in the Results section and diagrammatically depicted in Fig. 1. The modified cDNAs were excised from pUC57 and inserted into the modified pUC19 vector. Finally, the full length cDNA was sub-cloned into a pMPG-CR5 expression vector^26^ for transfection into CHO-BRI-rcTA cells and protein production. Proteins were collected from the supernatants of transfected cells and analyzed by SDS-PAGE and Western blotting with antibodies to the α and β subunits of the IGF-1R and the Fc domain of human IgG_1_, as described^26^, to determine purity and the presence of oligomerized HMW species.

### Generation of cell lines stably producing IGF-TRAPs

Two pools of CHO-BRI-rcTA-55E3 cells^27^ stably expressing IGF-TRAP-3.1 or IGF-TRAP-3.3 were generated. Briefly, the cells were transfected with pMPG-CR5-IGF-TRAP-3.1 or pMPG-CR5-IGF-TRAP-3.3 using electroporation and cultured in PowerCHO2 chemically defined medium (Lonza, Walkersville, MD) supplemented with 4 mM L-glutamine. Hygromycin B (650 μg/ml) was used for selection of stably transfected cells. To obtain clonal populations stably expressing the IGF-TRAP 3.3, the pooled cells were plated in a CloneMatrix semi-solid medium (Molecular Devices, Menlo Park, CA) at a density of 600–800 cells/ml and single colonies isolated 7–8 days later using the CellCelector™ device (Aviso, Jena, Germany). The colonies were expanded and their supernatants analyzed by polyacrylamide gel electrophoresis (PAGE) to confirm production of the modified IGF-TRAPs.

### Production and purification of modified IGF-TRAPs

Cells stably producing the IGF-TRAPs were cultured in *BalanCD*™ *CHO Growth* A medium at 37 °C in shaker flasks or wave bags to a density of 3–4 × 10^6^ cells/ml. IGF-TRAP production was induced by the addition of cumate (3 μg/ml) to the medium and a temperature shift to 32 °C. Feed B or Feed 12.7 supplements (Irvine Scientific, Santa Ana, CA) were added to the culture medium during production, as per the manufacturer’s instructions. When cell viability reached 70% or less, the supernatants were harvested, IGF-TRAP protein purified using a Protein A MabSelect SuRe resin (GE Healthcare Mississauga, ON) and frozen at −70 °C in a buffer containing 12.5 mM Na_3_PO_4_, 150 mM NaCl, 0.01% Tween 80 at pH 7.2.

### Production and purification of dusigitumab (MEDI-573)

The cDNAs encoding the heavy (HC) and light (LC) chains of dusigitumab (or MEDI-573; KEGG drug entry D10352; PubChem 172232445) were synthesized *de novo* with N-terminal signal peptide (MPLLLLLPLLWAGALA for HC; MRLPAQLLGLLMLWVSGSSG for LC) by Geneart (Thermo Fisher Scientific, Waltham, MA) using CHO codon bias, and cloned in the pTT96™ stable expression vector^27^. A stable pool of MEDI-573-producing CHO cells was generated and the protein produced purified from the supernatant using protein-A chromatography, as described^27^.

### *In vitro* binding affinity measurements

Ligand binding to the modified IGF-TRAPs was measured using a BioRad Proteon (BioRad Inc., Mississauga, ON, Canada) surface plasmon resonance (SPR) instrument. GLC sensorchips, the Biorad ProteOn amine coupling kit (EDC, sNHS and ethanolamine), and 10 mM sodium acetate buffers were purchased from Bio-Rad Laboratories (Mississauga, ON, Canada). PBS running buffer with 0.05% Tween 20 (PBST) was purchased from Teknova Inc. (Hollister, CA). Goat polyclonal anti-human Fc antibody was purchased from Jackson Immunoresearch Laboratories Inc. (West Grove, PA). All IGF binding experiments were carried out with PBST running buffer at a temperature of 25 °C. The anti-human Fc capture surface was generated using sNHS/EDC, as recommended by the manufacturer until 4000 RUs were immobilized. The screening of IGF-TRAP binding to hIGF-1, hIGF-2 or mIGF-1 occurred in two steps: (i) an indirect capture of the IGF-TRAP onto the anti-human Fc antibody surface followed by (ii) the simultaneous injection of 5 concentrations of purified ligand and one buffer blank for double referencing in the analyte direction. For each IGF-TRAP, approximately 1200 RUS were captured onto the anti-hFc surface by injecting 10 μg/ml solutions for 300 s at a rate of 10 μl/min. This step was immediately followed by two buffer injections to stabilize the baseline, and then the IGF ligands, along with a buffer blank were simultaneously injected at 50 μl/min for 120 s with a 600 s dissociation phase. Serially diluted ligands (320–3.95 nM) were used and each dilution injected in triplicates. The captured IGF-TRAP surfaces were regenerated by an 18 s pulse of 0.85% phosphoric acid for 18 s at 100 μl/min to prepare for the next injection cycle. Sensorgrams were aligned and double-referenced using the buffer blank injection and interspots of anti-Fc-only and analyzed using ProteOn Manager Software v3.1. The double-referenced sensorgrams were then fit into the 1:1 binding model.

Due to the complexity of the IGF – IGF-1R interaction, competition binding experiments were also carried out to assess solution binding affinity. A Biacore T200 (GE Healthcare, Mississauga, ON, Canada) was used to carry out the experiments at 25 °C using PBS containing 0.05% Tween 20 as running buffer (Teknova, Hollister, CA). Approximately 4000 resonance units (RUs) of the MEDI-573 antibody were immobilized onto the surface of a CM-5 sensorchip using the standard amine coupling within the Immobilization Wizard application, according to the manufacturer’s protocol. Five nM of hIGF-1 or hIGF-2 (Sigma-Aldrich, Oakville, ON, Canada) were incubated with increasing amounts of IGF-TRAP (0.12–500 nM) and after a 1 h equilibration, the mixtures were injected in duplicates over the MEDI-573-bound surface and the mock-activated (referencing) surface for 60 s at 30 μl/min. The MEDI-573 surfaces were regenerated with a 30 s injection of 10 mM glycine pH 1.5. Sensorgrams were double referenced and the free ligand binding on the antibody surface measured 10 s after the end of injection. The IC50 values were calculated using GraphPad Prism 7.04 by normalizing and fitting the competition data to a variable slope 4 parameter fit.

IGF-TRAP binding to the neonatal Fc receptor (FcRn) was also measured using the BioRad Proteon instrument. Five-thousand RUs of a 20 μg/ml solution of NeutrAvidin (Thermo-Fisher, Burlington, ON, Canada) in 10 mM Na acetate, pH 4.5 were coupled to two flow channels of a GLC sensorchip using standard NHS/EDC methods with one channel being used as a blank reference and the other for capture of 80 RUs units of FcRn biotinylated at the C-terminus using a BAP-TAG (produced internally). Serial dilutions of IGF-TRAP-3.1 and IGF-TRAP-3.3 were injected over the immobilized FcRn at a flow rate of 50 μl/min for 120 s with 180 s for dissociation. Sensorgrams were double-referenced using the NeutrAvidin blank surface and fit to steady-state fits using the Proteon Manager Software v3.1.

### Pharmacokinetic analysis

C57Bl/6 and Balb/c female mice were obtained from Charles River Laboratories (St. Constant, QC, Canada). Female Ncr Nude mice were obtained from Taconic Biosciences (Rensselaer, NY). The mice were used in these experiments at the age of 7–12 wk old. Mice were injected intravenously (i.v.) with 10 mg/kg IGF-TRAP. They were then divided into several groups of 3 mice each and blood collected from alternate groups, beginning at 5 min and up to 10 days post injection. Plasma IGF-TRAP levels were analyzed using the soluble IGF-1R ELISA Kit (R&D Systems, Minneapolis, MN). Data are a pool of each group of mice bled at the same time interval. Non-compartmental data analysis and pharmacokinetic parameters were determined using Phoenix WinNonlin Software v6.2 (Pharsight Corporation, St. Louis MO). The area under concentration versus time curve from the start of dose administration to the last observed quantifiable measure (*AUC*_*0-last*_) was estimated and total body clearance (CL) was calculated by dividing the intravenous dose by the area under concentration versus time curve from the start of dose administration to infinity.

### Bio-distribution analysis

The bio-distribution of the IGF-TRAP in C57BL/6 mice was analyzed using ^111^In-labelled IGF-TRAP that was detected *in situ* by whole animal imaging using single-photon emission computed tomography (SPECT). The radio-conjugate TRAP-DOTA (^111^In-DOTA-IGF-TRAP) was generated using the protocol previously described^28^ to obtain a specific activity of ≥1.2 μCi/μg and radiochemical purity of >99% at the time of injection that was generally administered within 24 h of radiolabeling. A dose of 30 μCi/animal (25 μg conjugate, ~1 mg/kg) was found to give an adequate signal in SPECT with an acquisition time of 30 min per animal. The animals were imaged repeatedly over a few days, sacrificed 72–96 h post injection and their organs harvested and analyzed for uptake of radioactivity. The SPECT images were processed and analyzed by the VivoQuant 2.5 (InviCRO) software. Uptake in the liver was normalized to activity in blood sampled immediately after tail vein injection.

For *ex-vivo* tissue bio-distribution analyses, different groups of animals were injected i.v. with ^111^In-DOTA-IGF-TRAP at specific activities of ~0.6 μCi/μg (15 μCi/animal, injected at a dose of 1 mg/kg). At the indicated time intervals post IGF-TRAP injection, the mice were anaesthetized, blood withdrawn by cardiac puncture and organs harvested. Where indicated, the livers were perfused with saline followed by 0.4% paraformaldehyde (PFA) to remove residual blood, and liver fragments were collected. Organs and blood were weighed and the radioactivity associated with each measured in a gamma counter (Wizard 2470, ^111^In counting efficacy-26.9%). Counts were calculated as μCi/μg tissue or %ID/g, or tissue/blood ratios (to normalize against variation in the injection) taking into account radioisotope decay and counting efficacy.

### The kinase IGF-1 receptor (KIRA) assay

The KIRA assay was used to assess residual ligand bioactivity in the circulation of IGF-TRAP-treated mice and performed as described in detail elsewhere^19,29^ including recent modifications^30^. In brief, HEK293 cells overexpressing IGF-1R were cultured in complete DMEM medium (containing 1% Penicillin/Streptomycin, 0.5 mg/ml G418, 0.25 mg/ml Hygromycin, 10% FCS). The cells were then seeded in a 48-well microtiter plate (4 × 10^5^ cells per well) for 24 h with serum, followed by a 24 h incubation without serum. After 48 h, sera from mice injected with 0.5 or 10 mg/kg IGF-TRAP diluted (1:10) in Krebs Ringer buffer or serially diluted rhIGF-1 (concentrations ranging from 0.3125–10 ng/ml, for standard curve) were added to the cells. Following a 15 min incubation at 37 °C to activate the IGF-1R, the cells are lysed (in HEPES lysis buffer) and the lysate transferred to a microtiter plate pre-coated with an IGF-1R capture antibody (Human phospho-IGF-1R ELISA kit, R&D Systems, Minneapolis, MN) and probed with an HRP-conjugated anti-phospho-tyrosine antibody. IGF-1R activation levels were measured at a wave length of 450 nm. Area under the IGF-1 serum concentration vs time curves up to day 18 were calculated using Phoenix WinNonlin Software v8.0.0.3176 (Pharsight Corporation).

### Experimental metastasis assays

Female C57Bl/6 mice were inoculated via the intrasplenic/portal route with 5 × 10^4^ MC-38 followed by splenectomy, as previously described^5,31^. IGF-TRAP or Mab MEDI-573 injections were administered i.v. at the indicated dose on day 1 post tumor cell inoculation and on alternate days thereafter until day 10. Mice were sacrificed 16 days post tumor inoculation, livers resected and visible metastases on the surfaces of the livers enumerated immediately before fixation. Some of the livers were fixed in 10% phosphate buffered formalin, paraffin-embedded, and 4 μm paraffin sections cut and stained with hematoxylin and eosin to visualize micrometastases.

### Statistical analysis

Metastasis data were analyzed by the non-parametric Mann-Whitney test and all other data by a one-tailed student t-test.

## Results

### Characterization of variant IGF-TRAPs

We previously reported on the production of an IGF-TRAP with potent anti-cancer effects. We documented that in the IGF-TRAP preparations, HMW protein species that migrated at a >400 kDa range could be minimized by step elution following Protein-A column purification. The fractions eluted at pH 3.5 that was enriched for HMW proteins had a markedly reduced ligand affinity and a shorter than expected half-life of only 10 hours, indicating that the HMW species did not contribute significantly to the biologic activity of the IGF-TRAP. HMW species of Fc-fusion proteins have been observed by others and generally attributed to oligomerization due to the formation of irregular disulfide bonds between individual Fc domains^32^. In an effort to further improve the purity and manufacturability of the IGF-TRAP, we therefore undertook to modify the parent protein, such that the HMW species would be minimized.

**Figure 2:**
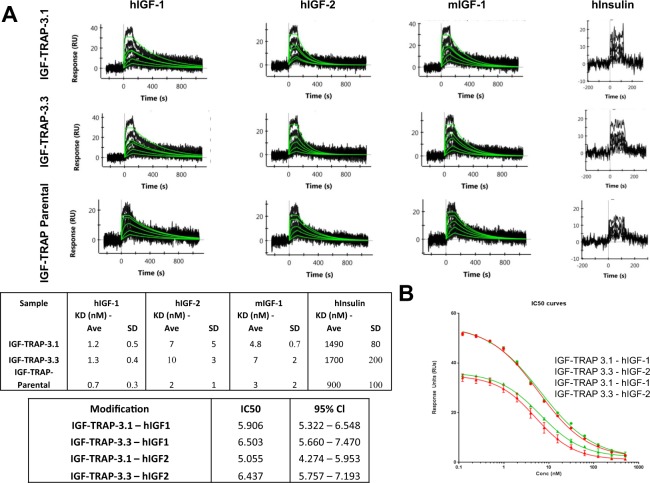
SPR direct binding and competition assays show similar ligand binding affinities for IGF-TRAP 3.1 and IGF-TRAP 3.3. Direct ligand binding was measured by first capturing the IGF-TRAP onto an immobilize anti-Fc surface (4000 resonance units (RUs)), and then flowing a dilution series of the ligands indicated over the captured IGF-TRAP. Shown in (A-top) are results of binding sensorgrams of a representative analysis of a total of 3 performed. The kinetic fits used for affinity determination are shown in green. The calculated affinity constants (mean ± SD, n = 3) based on a fit to a 1:1 Langmuir binding model are shown in the Table (**A**-bottom). Shown in (**B**) are results of a binding competition assay in which increasing concentrations of IGF-TRAP-3.1 or IGF-TRAP-3.3 were used to compete with binding of constant ligand concentrations to MAb MEDI-573-immobilized onto the SPR surface. The SPR response of IGF binding to MEDI-573 is shown (B-top) with IGF-TRAP-3.1 (in red), IGF-TRAP-3.3 (in green) and using rhIGF-1 (circles) or rhIGF-2 (triangles) as ligands. Shown in (B-bottom) are IC50 values for IGF-TRAP-3.1 and IGF-TRAP-3.3 with the 95% CI (in parentheses) as determined based on the SPR competition binding assays.

Four different modified IGF-TRAP constructs were generated: (i) the two cysteines in the core hinge region of the human IgG_1_ Fc fragments were substituted with serines (IGF-TRAP-3.1); (ii) the 11-aa artificial linker EFDISSTMVRS between each extracellular domain of human IGF-1R ectodomain β-chain and the human IgG_1_ Fc fragment were replaced with a 22-aa (G_4_S)_4_G_2_ flexible linker (IGF-TRAP-3.2); (iii) both of the above modifications were introduced (IGF-TRAP-3.3); and (iv) the 11-aa artificial linker, the 5-aa upper hinge region and the first 3-aa of the core hinge region (including the first Cys) of the Fc fragment were replaced with a slightly longer (27-aa) flexible linker (G_4_S)_5_G_2_ (IGF-TRAP-3.4) (Fig. 1A and Supplementary Fig. S1). Considering that the distance between the two C-termini of the IGF-1R ectodomain (αβ)_2_ heterotetramer β-chains is ~120 Å, and the distance between the two N-termini of the Fc homodimer hinge region is ~10 Å, we estimated that each of the two linkers of the IGF-TRAP will have to span a linear distance of ~55 Å (see Supplementary Fig. S1), which can be reasonably achieved by a flexible linker of 22 amino-acid residues^25^. We reasoned that with this longer and more flexible linkage in IGF-TRAPs 3.2 and 3.3, the 3D structures of the IGF-1R ectodomain (αβ)_2_ heterotetramer and the Fc homodimer would be preserved within an IGF-TRAP tetrameric unit. On the other hand, for the shorter and less flexible linkage that remained in IGF-TRAP 3.1, the Fc hinge regions may be structurally stretched and act as additional linkers in order to fill the gap required for linkage of the 2 units. While in the parental IGF-TRAP construct, such hinge distortions could have resulted in exposure of cysteine residues and disulfide shuffling with the resulting formation of higher MW oligomers, we reasoned that this could be corrected in IGF-TRAP 3.1 (and to some extent in IGF-TRAP 3.3) by substituting the cysteine residues with serines. Because the IGF-TRAP-3.4 variant retained only one of the two cysteines in the Fc fragment due to N-terminal truncation of the hinge region, a slightly longer flexible linker of 27-aa was utilized. PAGE analysis of the modified IGF-TRAPs (Fig. 1B,C) confirmed the reduction in HMW species to undetectable levels in both IGF-TRAPs 3.1 and 3.3, but not in IGF-TRAPs 3.2 and 3.4. This indicated that the observed HMW species were indeed likely due, at least in part, to disulfide bonding between adjacent Fc homodimers. SPR analysis (Fig. 2A) confirmed that both IGF-TRAP-3.1 and IGF-TRAP-3.3 retained the high affinity binding of the parent IGF-TRAP to both human and mouse IGF ligands, while also exhibiting a 10^3^-fold lower affinity for human insulin. This was confirmed in a binding competition assay, using a constant hIGF concentration of 5 nM and increasing concentrations of the IGF-TRAPs (Fig. 2B), with MAb MEDI-573 immobilized on the SPR surface.

### Pharmacokinetic analysis reveals increased stability of IGF-TRAP-3.3 in the circulation

**Figure 3:**
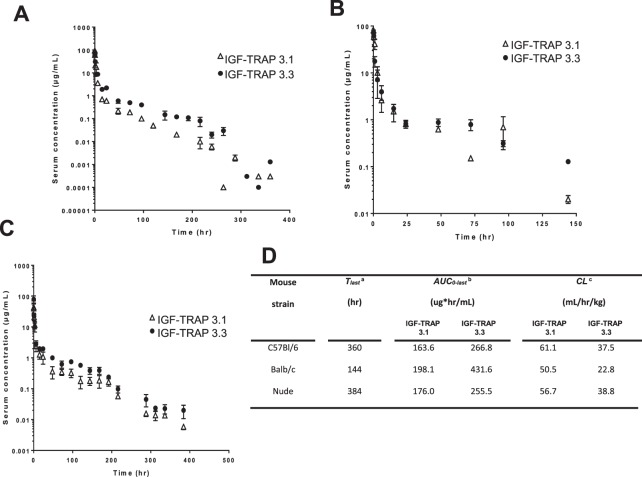
Pharmacokinetic analysis reveals a more rapid clearance of IGF-TRAP 3.1 from the circulation following bolus i.v. injections in three mouse strains. C57Bl/6, Balb/c and nu/nu female mice were tail-vein injected with 10 mg/kg of the indicated IGF-TRAPs. Blood was collected at the indicated time intervals for measurement of circulating IGF-TRAP proteins in the serum using soluble IGF-1R ELISA assay. Data are based on 3 mice bled at the same time intervals. Shown in (**A**–**C**) are mean serum concentrations values (±SEM) for C57Bl/6, Balb/c and nu/nu mice respectively. Shown in (**D**) are calculated clearance and area under the serum concentration-time curve values determined using the Phoenix Win-Nonlin Software v6.2. ^a^T_last_ – time of the last measured plasma concentration. ^b^AUC_0-last_ – area under the concentration-time curve up to the last measured plasma concentration. ^c^CL – total body clearance.

We next assessed the effect of the molecular modifications on the pharmacokinetic properties of IGF-TRAPs 3.1 and 3.3 in preparation for evaluating their biological activity. Mice were injected intravenously with 10 mg/kg IGF-TRAP and serum concentrations measured at intervals from 5 min to 7 days post injection, as we previously described^15^. Shown in Fig. 3 are serum concentration-time profiles for the two IGF-TRAPs injected into C57Bl/6, Balb/c and nu/nu mice. For both, we observed a sharp drop in serum concentrations within the first 15 h post injection, at which time the concentrations stabilized. A non-compartmental pharmacokinetic analysis (Fig. 3D) revealed clearance values ranging from 50.5 to 61.1 ml/hr/kg for IGF-TRAP-3.1, and from 22.8 to 38.8 ml/hr/kg for IGF-TRAP-3.3, indicating that the addition of a flexible linker altered the stability of the latter in the circulation. Our data thus showed that following administration of 10 mg/kg intravenously, the area under the serum concentration-time curve was 63%, 118% and 45% higher for IGF-TRAP-3.3 as compared to IGF-TRAP-3.1 in all three strains namely C57Bl/6, Balb/c and immunocompromised nude mice, respectively.

### Bio-distribution analysis reveals initial retention of the IGF-TRAPs in the liver

**Figure 4:**
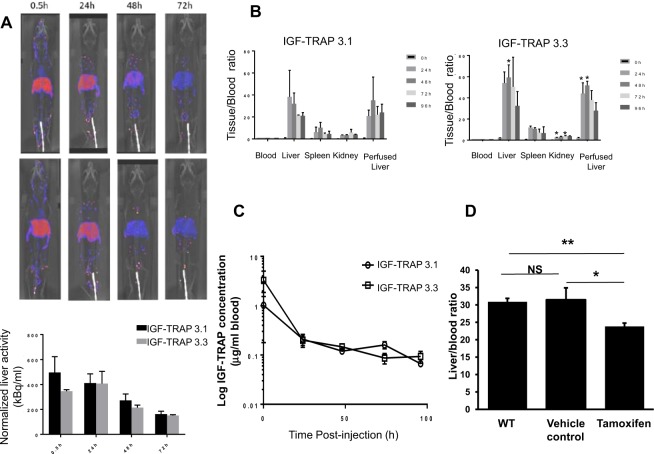
Tissue biodistribution of IGF-TRAP 3.1 and IGF-TRAP 3.3. SPECT/CT was performed on mice injected i.v. with ^111^In-DOTA-IGF-TRAP. Shown in (**A**) are representative SPECT/CT images of mice injected with IGF-TRAP 3.1 (top panels) and IGF-TRAP 3.3 (bottom panels), and below are the averages (±SD) of liver activity normalized to dose (n = 2). Shown in (**B**) are IGF-TRAP radioactivity values in resected organs as measured by a gamma counter and expressed as a mean ratio to radioactivity measured in the blood (±SD) (n = 3). Shown in (**C**) are blood concentrations of the indicated radiolabeled IGF-TRAPs following a bolus i.v. administration. Results are expressed as means (±SD) based on 3 animals. To compare IGF-TRAP 3.3 retention in iLID and control mice (**D**), 25 μg of IGF-Trap3.3-DOTA-In^111^ with a specific activity of 0.6 μCi/μg were injected into C57BL/6 mice and into iLID mice that received an i.p. injection of 0.3 mg tamoxifen in sunflower oil or oil alone (vehicle control) 8 days earlier. Livers were resected and blood collected 72 hr later and radioactivity measured in a γ counter. Shown are IGF-TRAP radioactivity values in the livers expressed as a mean ratio to radioactivity measured in the blood (±SEM, n = 3). *p ≤ 0.05, **p ≤ 0.003, NS-not significant.

The rapid decline in serum IGF-TRAP concentrations following i.v. injection pointed to extra-vascular tissue retention of the IGF-TRAP proteins. To analyze their bio-distribution, we used ^111^In-conjugated IGF-TRAPs and monitored the retention of radioactivity in different organs post i.v. IGF-TRAP injection *in vivo* using SPECT/CT imaging. Our results, shown in Fig. 4, revealed a rapid and preferential retention of both IGF-TRAPs in the liver, with low levels also detectable in the kidneys and spleen (Fig. 4A). This was also confirmed by *ex vivo* analysis of resected organs using a gamma counter (Fig. 4B). Although a slight (but significant) increase in the retention of IGF-TRAP 3.3 in the liver was seen at the early time points (24 h and 48 h) post injection, overall the bio-distribution patterns were similar. For both IGF-TRAPs, serum concentrations at equilibrium (24 h post injection) represented approximately 6% of the total IGF-TRAP injected, measured as total radioactivity in the circulation (Fig. 4C). These values corresponded to serum concentration/time profiles obtained using the ELISA assay and indicated that the sharp initial drop in serum IGF-TRAP concentrations was due to rapid capture and retention of the IGF-TRAP in the liver.

The liver is the major source of plasma IGF-1. To determine whether the retention of the IGF-TRAPs in the liver was due to preferential ligand binding in this organ, we compared IGF-TRAP 3.3 accumulation in livers of wild type mice and mice with a conditional, liver-specific, IGF-I deficiency (iLID) induced by a single tamoxifen injection^3,20^. Previously, we have shown that in these mice, IGF-1 levels decline by up to 70% within 18 hr of a tamoxifen injection^3,20^. When the ^111^In-labelled IGF-TRAP 3.3 was injected into these mice, we found a 23% reduction in the liver/ blood concentration ratio of the TRAP in these mice (as measure by a γ counter) relative to controls (Fig. 4D), suggesting that ligand binding contributed to the retention of the IGF-TRAP in the liver.

### Both IGF-TRAPs inhibit experimental colon carcinoma metastasis but display distinct dose response kinetics

**Figure 5:**
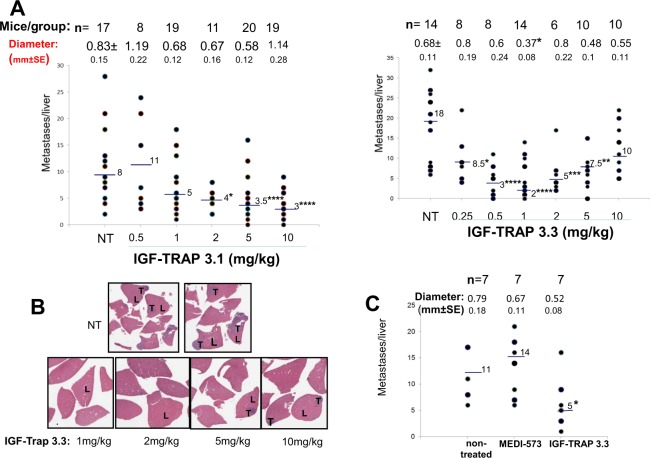
Distinct dose response kinetics for the inhibitory effects of IGF-TRAP 3.1 and IGF-TRAP 3.3 on experimental colon carcinoma liver metastasis. Mice were injected with 5 × 10^4^ MC-38 cells, via the intrasplenic/portal route. Intravenous IGF-TRAP injections at the indicated doses began one day later and continued twice weekly, for a total of 5 injections. Mice were sacrificed 16–18 days post tumor cell inoculation and visible liver metastases on the surfaces of the livers were enumerated. Shown in (**A**) are the numbers of metastases counted per individual livers in 3 different experiments combined. Horizontal bars denote medians. The total number of mice per treatment group is indicated in the top row and the mean nodule size per group (in mm ± SE) below. Shown in (**B**) are representative H&E stained formalin fixed paraffin embedded sections from several livers per each of the indicated treatment groups for which metastases data are shown in (**A**). Shown in (**C**) are results of a separate experiment where one group of mice was treated with 1 mg/kg of the anti-ligand MAb MEDI-573. Horizontal bars denote medians. (L) – liver; (T) – tumor; NT – non-treated. *p < 0.05, **p < 0.01, ***p < 0.005, ****p < 0.001, as assessed by the Mann-Whitney non-parametric test.

The liver is the major site of IGF-1 production, and we and others have previously shown that liver derived IGF-1 promotes the growth of hepatic metastases^1,33^. To evaluate the effect of the newly engineered IGF-TRAPs on the growth of colon carcinoma liver metastases, mice were inoculated with (5 × 10^4^) murine colon carcinoma MC-38 cells via the intrasplenic/portal route and subsequently injected intravenously from day 1 post tumor injection with the IGF-TRAPs at doses ranging from 0.5–10 mg/kg. Mice were sacrificed 16–18 days post tumor inoculation and liver metastases enumerated. In mice treated with either one of the IGF-TRAPs, we observed a dose-dependent response to IGF-TRAP treatment, as evidenced by significant reductions in the number and size of visible hepatic metastases (Fig. 5A) and also confirmed by hematoxylin and eosin (H&E) staining of formalin fixed and paraffin embedded liver sections (Fig. 5B). However, whereas the inhibitory effect of IGF-TRAP-3.1 was optimal at doses of 5–10 mg/kg, thereby replicating the dose-response kinetics of the parental IGF-TRAP^15^, IGF-TRAP-3.3 reduced experimental liver metastasis most effectively at doses of 0.5–1 mg/kg, with no added therapeutic benefit at higher doses of up to 10 mg/kg (Fig. 5A). Significantly, at the dose of 1 mg/kg, IGF-TRAP-3.3 had a superior therapeutic effect to that of the anti-ligand antibody (MEDI-573), now in phase II clinical trials, as assessed in the experimental colon cancer liver metastasis model (Fig. 5C).

### The KIRA provides a surrogate biomarker for IGF-TRAP efficacy

**Figure 6:**
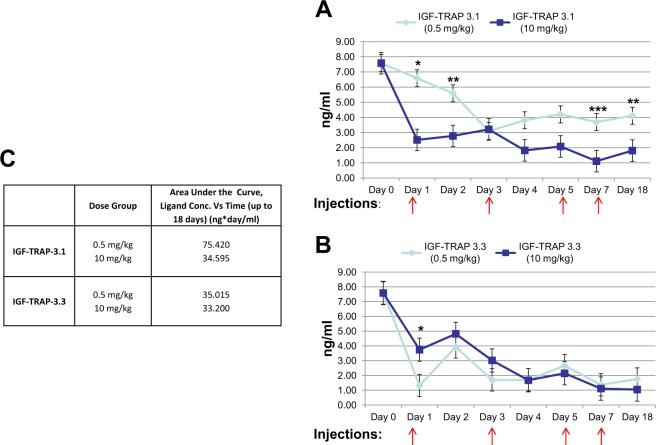
The KIRA reveals dose dependent differences in the abilities of IGF-TRAPs 3.1 and 3.3 to reduce the bioavailability of circulating IGF-1. Blood was collected daily from mice that underwent injections with IGF-TRAP 3.1 (**A**) or IGF-TRAP 3.3 (**B**) at doses of 0.5 mg/kg or 10 mg/kg on alternate days (as indicated by arrows). Shown in (**A**,**B**) are plasma levels of bioavailable IGF-1, as measured by the KIRA for the indicated time intervals, and in (**C**) are the concentration/time ratios calculated based on the data. *p < 0.05, **p < 0.01, ***p < 0.001.

One obstacle to the clinical utility of IGF targeting drugs has been the lack of reliable biomarkers for prediction of response and patient selection. We asked whether serum IGF bioavailability correlated with the IGF-TRAP pharmacodynamic profile and could predict the response of the animals to IGF-TRAP treatment. To this end, we used the KIRA assay to measure ligand bioavailability following treatment with different doses of IGF-TRAP-3.1 and IGF-TRAP-3.3. This method quantifies IGF-IR activation levels rather than total plasma IGF concentrations (bound and unbound) and therefore more accurately reflects the net bioactivity remaining in the serum of IGF-TRAP treated mice. Mice were treated with 0.5 and 10 mg/kg of each of the IGF-TRAPs, corresponding to the optimal therapeutic ranges observed for IGF-TRAP 3.3 and IGF-TRAP 3.1, respectively, in the experimental liver metastasis model. Injections were administered on alternate days for 10 days to mimic the therapeutic protocol and blood collected daily to monitor changes in serum IGF-1 bioavailability. In agreement with the therapeutic profiles of these IGF-TRAPs, we observed a maximal reduction in IGF-1 bioavailability using a dose of 10 mg/kg for IGF-TRAP-3.1 (Fig. 6A) and of 0.5 mg/kg for IGF-TRAP-3.3 (Fig. 6B), and this was also reflected in distinct AUC values for each IGF-TRAP (Fig. 6C), suggesting that the pharmacodynamic properties of these IGF-TRAPs corresponded to their ability to reduce the bioavailability of serum IGF-1.

## Discussion

We bioengineered four novel IGF-TRAP variants and two (i.e. IGF-TRAPs 3.1 and 3.3) were selected for further analysis based on markedly reduced protein oligomerization and improved manufacturability. Importantly, the modifications engineered into IGF-TRAPs 3.1 and 3.3 did not affect the ligand binding affinities of either, as the *in vitro* measured *K*_D_ of both remained consistent with the values obtained for the parent IGF-TRAP. However, a difference was observed in the pharmacokinetic and pharmacodynamic properties of these IGF-TRAPs. We previously reported that in an experimental model of colon cancer metastasis, using murine colon carcinoma MC-38 cells, the parent IGF-TRAP reduced the median number of liver metastases by 32, 49 and 59% respectively, at the doses of 2, 5 and 10 mg/kg^15^. Using the same injection regimen, we observed similar dose response kinetics with IGF-TRAP 3.1, with reductions of approximately 50, 54 and 62% respectively, in the median number of liver metastases at the same doses. Thus, the biological response to IGF-TRAP 3.1 mimicked that of the parent IGF-TRAP, although it had a moderately increased efficiency, particularly at the low doses, likely due to improved purity. In contrast, however, both the serum retention properties, therapeutic efficacy and dose response kinetics of IGF-TRAP-3.3 were altered, as the optimal anti-metastatic effect in the colon carcinoma model was observed at the 10-fold lower doses of 0.5–1 mg/kg. This improved therapeutic profile was likely the consequence of the addition, in IGF-TRAP-3.3, of an extended flexible linker between the IGF-1R ectodomain β-chains and the hinge regions of the Fc homodimer in order to replace the shorter and more rigid linkers of IGF-TRAP-3.1 – the only known structural difference between these two IGF-TRAPs. The length and flexibility of a linker have previously been shown to affect the stability, affinity and functionality of antibody fragments^34^. We considered the possibility that the shorter and more rigid linker in IGF-TRAP 3.1 alters its binding to the neonatal Fc receptor (FcRn) via the Fc domain and thereby its recycling. Under physiological conditions, the Fc domain binds FcRn at the acidic pH of the early endosomes and is released at the extracellular neutral pH. FcRn has been shown to protect bound proteins from intracellular degradation, thereby prolonging circulation time in the plasma and modulating systemic clearance^16,35^. However, in standard SPR experiments, we found that the binding affinities and kinetic parameters of both IGF-TRAPs to the human FcRn ectodomain were very similar when evaluated under acidic pH (Supplementary Fig. S2). It should be noted that under similar conditions, indistinguishable FcRn binding properties were also observed for antibodies and Fc fusion proteins that displayed large differences in their pharmacokinetic properties^36^. While we cannot, at present, completely rule out the possibility that minor differences in binding properties may be better discernible under different pH conditions^37–39^, our results suggest that differential FcRn binding does not play a major role in the distinct pharmacokinetic properties of these IGF-TRAPs. FcgR- binding to liver sinusoidal endothelial cells has recently been shown to accelerate the clearance of bioengineered antibodies^40^. Whether or not differences in clearance of IGF-TRAP 3.1 and IGF-TRAP 3.3 via this mechanism played a role in their distinct pharmacokinetic and pharmacodynamic profiles remains to be investigated.

The asialoglycoprotein receptors are highly expressed on the surface of the hepatocytes and can actively endocytose and degrade glycoproteins with exposed terminal galactose or N-acetylgalactosamine residues^41^. We also analysed terminal glycoconjugates on the two IGF-TRAPs using the SialiQuant Sialic Acid Quantitation Kit (QAbio, Cedarlane, Burlington, ON, Canada) in order to assess the potential contribution of glycoconjugates to their distinct properties. Although no significant differences in their total sialic acid contents were detected (data not shown), the possibility that more subtle or localized glycan differences between the IGF-TRAPs could have influenced their blood clearance by the asialoglycoprotein receptors, cannot be ruled out.

The mechanism(s) mediating the high retention of the IGF-TRAPs in the liver remain to be fully elucidated. We observed a reduction in the accumulation of IGF-TRAP 3.3 in the livers of iLID mice where liver IGF-1 production is abolished. This suggests that some of the liver-specific retention was probably due to ligand binding. However other mechanisms are likely to also be involved because the reduction we observed was partial (23%). It is possible that sialic acid residues on the IGF-TRAPs also contribute to this retention by mediating binding to the asialoglycoprotein receptors expressed on the surface of the hepatocytes.

In our therapeutic studies, we observed a potent inhibitory effect of IGF-TRAP-3.3 on liver metastasis when mice were administered twice weekly with 0.5 and 1 mg/kg, and this effect stabilized and even slightly declined when higher doses of up to 10 mg/kg were used, resulting in a U-shaped dose-response curve for this variant. This was not the case for IGF-TRAP-3.1. Importantly, these differences corresponded to the results of the KIRA analysis, where the reduction in bioavailable plasma IGF-1 was significantly greater following injection of 10 mg/kg than 0.5 mg/kg for IGF-TRAP-3.1, whereas the reverse was the case for IGF-TRAP-3.3, at least during the first 3 days following injection. This confirms the utility of the KIRA assay as a predictor of drug efficacy *in vivo* and also indicates that the difference in the rate of plasma clearance of the two IGF-TRAPs corresponded to real differences in their biological/therapeutic activity. U-shaped curves (also known as hormesis) have been observed in both clinical and preclinical studies of other cancer therapeutics, including chemotherapeutic drugs^42,43^ and biological agents such as cytokines^44,45^, endostatin^46^ and peptides^47^. Even monoclonal antibodies such as the anti-VEGF antibody Bevacizumab, that is presently in clinical use, showed better activity at lower doses^48,49^.

Several mechanisms have been proposed to explain U-shaped dose-response curves. They include (i) saturation effects, where targeted receptors are down-regulated when exposed to high concentrations of an inhibitor and this results in reduced ligand-binding capacity of the cells, (ii) increased clearance mechanisms and/or (iii) activation of feedback loops at high (but not low) inhibitor concentrations that can, in turn, modulate the concentrations and activity of a targeted ligand^47,49^. Physiological plasma IGF-1 levels are regulated via a feedback mechanism by pituitary derived growth hormone (GH). Reduced circulating IGF-1 levels trigger the release of GH, activation of GHR/STAT5 signaling in the hepatocytes and increased IGF-1 production, maintaining IGF-1 homeostasis^50^. We have previously shown that in mice with an inducible liver IGF-1 gene deletion (iLID mice), a sharp reduction in plasma IGF-1 levels, initiated by a tamoxifen injection, resulted in a rapid increase in circulating GH levels that compensated for loss of IGF-1 levels^3^. In mice treated with 0.5 or 10 mg/kg IGF-TRAP-3.3, we also observed a transient increase in plasma GH levels, but the kinetics were distinct, as injections of 0.5 mg/kg caused a sharp increase at 24 h which was of short duration, while injections of 10 mg/kg caused a delayed increase in GH levels that was, however, more sustained and lasted throughout the treatment period (Supplementary Fig. S3). This also corresponded to the sharper initial decrease in IGF-1 bioavailability that was detected by the KIRA following injection of 0.5 mg/kg, as compared to 10 mg/kg IGF-TRAP-3.3, although similar KIRA values were observed for both from day 4 onwards. These differences resulted in AUC values of 11.6 and 16.2 ng*day/ml between days 0 and 4, respectively following injections of 0.5 and 10 mg/kg IGF-TRA 3.3, although overall AUC values for the entire observation period (18 days) were similar. These findings suggest that at high IGF-TRAP-3.3 doses, a feedback loop resulting in increased IGF-1 production may have resulted in the loss of added therapeutic benefit and that the difference in IGF bioavailability in the first 4 days post tumor injections had profound effects on outcome. These results also identify the KIRA as a sensitive surrogate biomarker assay for gauging, predicting and calibrating the therapeutic response to IGF-TRAP treatment.

The disappointing performance of IGF-targeting drugs in clinical trials, despite documented highly beneficial effects in pre-clinical cancer models have led to calls for identifying biomarkers that could predict the clinical response to IGF inhibitors, thereby permitting a better patient stratification and individualized treatment. Our results suggest that at least for IGF-binding drugs, the KIRA assay could provide a sensitive tool to accompany evaluation of treatment efficacy, response prediction and dose calibration.

Finally, our results also highlight the need for proper protein engineering in the development of Fc fusion proteins. This has been well documented with the VEGF-TRAP, aflibercept, that required extensive protein engineering to improve the manufacturability and pharmacologic propertied of the drug^51^. Our results show that a structure-based step-wise development of a biological drug for improved manufacturability can also significantly improve pharmacological parameters and therapeutic efficacy.

## Electronic supplementary material

Supplemental Information
