# Supplementary material for: Enhanced anti-metastatic bioactivity of an IGF-TRAP re-engineered to improve physicochemical properties
Source: Sci Rep. 2018 Nov 26;8:17361. doi: 10.1038/s41598-018-35407-2 (PMC6255772; doi:10.1038/s41598-018-35407-2)
Supplement: Supplementary file 1 — Supplemental Information [file 41598_2018_35407_MOESM1_ESM.pdf]

Enhanced anti-metastatic bioactivity of an IGF-Trap re-engineered to improve physicochemical properties

George Vaniotis[1]*, Serge Moffett[1]*, Traian Sulea[4,6]* Ni Wang[1]*, S. Mehdy Elahi*[6], Etienne Lessard[6], Jason Baardsnes[6], Stephanie Perrino[1], Yves Durocher[6], Jan Frystyk[7], Bernard Massie[6] and Pnina Brodt[1,2,3,5,#&].

Departments of [1]Surgery, [2]Medicine and [3]Oncology, and [4]Institute of Parasitology, McGill University and [5]Cancer Research Program, Research Institute of the McGill University Health Center, Montreal Quebec, Canada, [6]Human Health Therapeutics Research Centre, National Research Council Canada, Montreal Quebec, Canada, and [7]Department of Clinical Medicine, Aarhus University, Aarhus, Denmark

<u>Running Title:</u> Distinct pharmacodynamic properties of bio-engineered IGF-TRAPs

* These authors contributed equally to this work.

[&] The authors of this manuscript have no competing financial or non-financial interests to report.

[#] **To whom all inquiries should be addressed at:** Research Institute of the McGill University Health Centre, 1001 Décarie Blvd, Glen Site, Room E.02.6220, Montréal, QC, H4A 3J1, Canada. Telephone: (514) 934-1934 (Ext. 36692) Fax: (514) 934-4460 Email: pnina.brodt@mcgill.ca

This study was supported by grant PP2-141724 from the Canadian Institute for Health Research, and grant IT04483 from MITACS (to PB) and by Amorchem Inc.

Supplementary Figure legends

Figure S1. A proposed 3D model of the IGF-TRAP. The extracellular domain of IGF-1R and the fused Fc domain of human IgG_1 are based on the known crystal structures of the ectodomain of hIR and the Fc domain of a $hIgG_1$, respectively.

Figure S2. IGF-TRAPs 3.1 and 3.3 bind to the FcRn with similar affinities. Shown are SPR sensorgrams of IGF-TRAP 3.1 and IGF-TRAP 3.3 flowing over 80 RUs of C-terminally biotinylated FcRn on a neutravidin sensor surface. The apparent K_D's based on an equilibrium fit gave similar results for each of the two IGF-TRAPs.

Figure S3. IGF-TRAP 3.3 injections alter circulating growth hormone (GH) levels. Blood was collected daily from mice injected with 0.5 or 10 mg/kg IGF-TRAP 3.3 on alternate days, as indicated. Shown are mean circulating GH levels (±SD) based on 3 animals per time point as measured by ELISA.

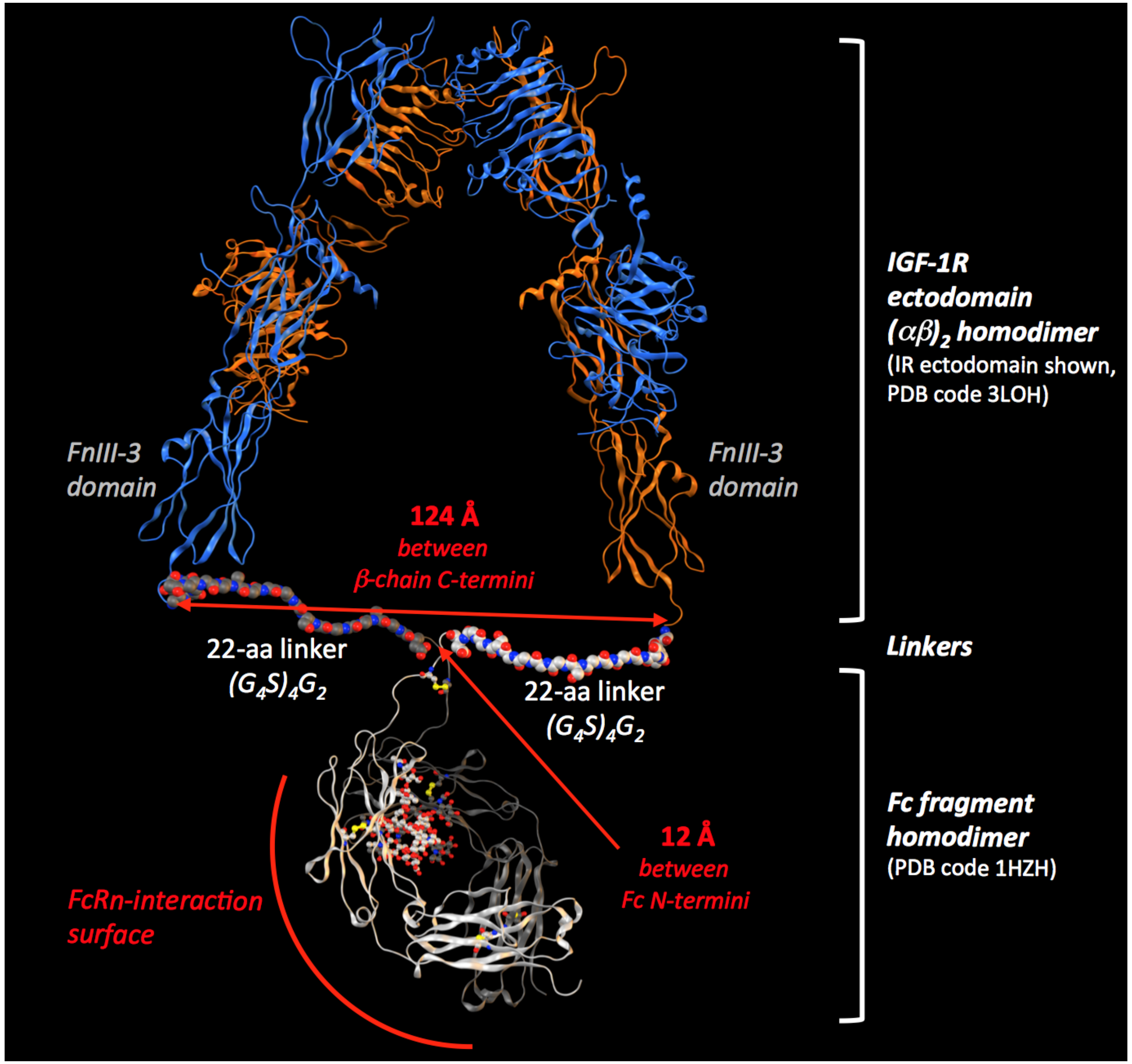

Supplementary Fig S1

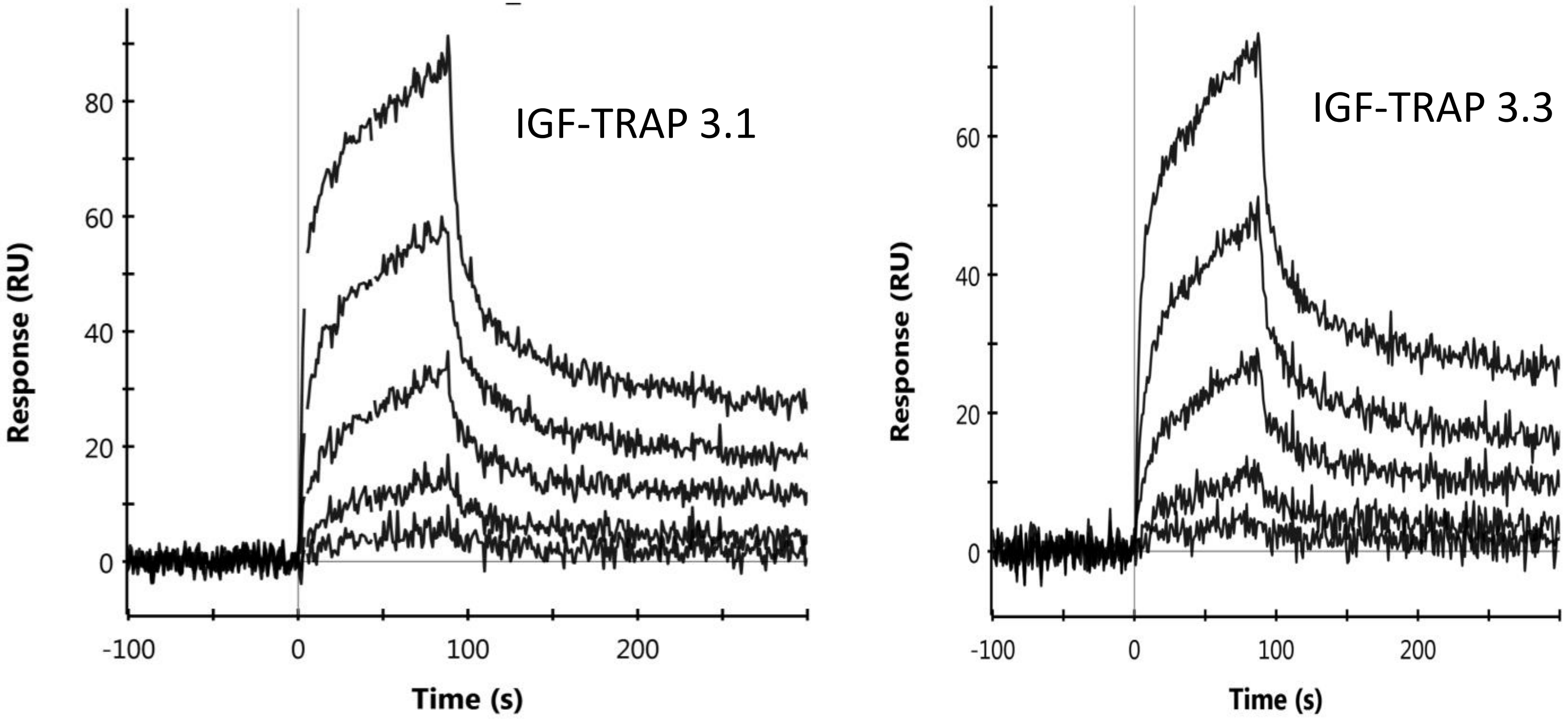

Supplementary Fig S2

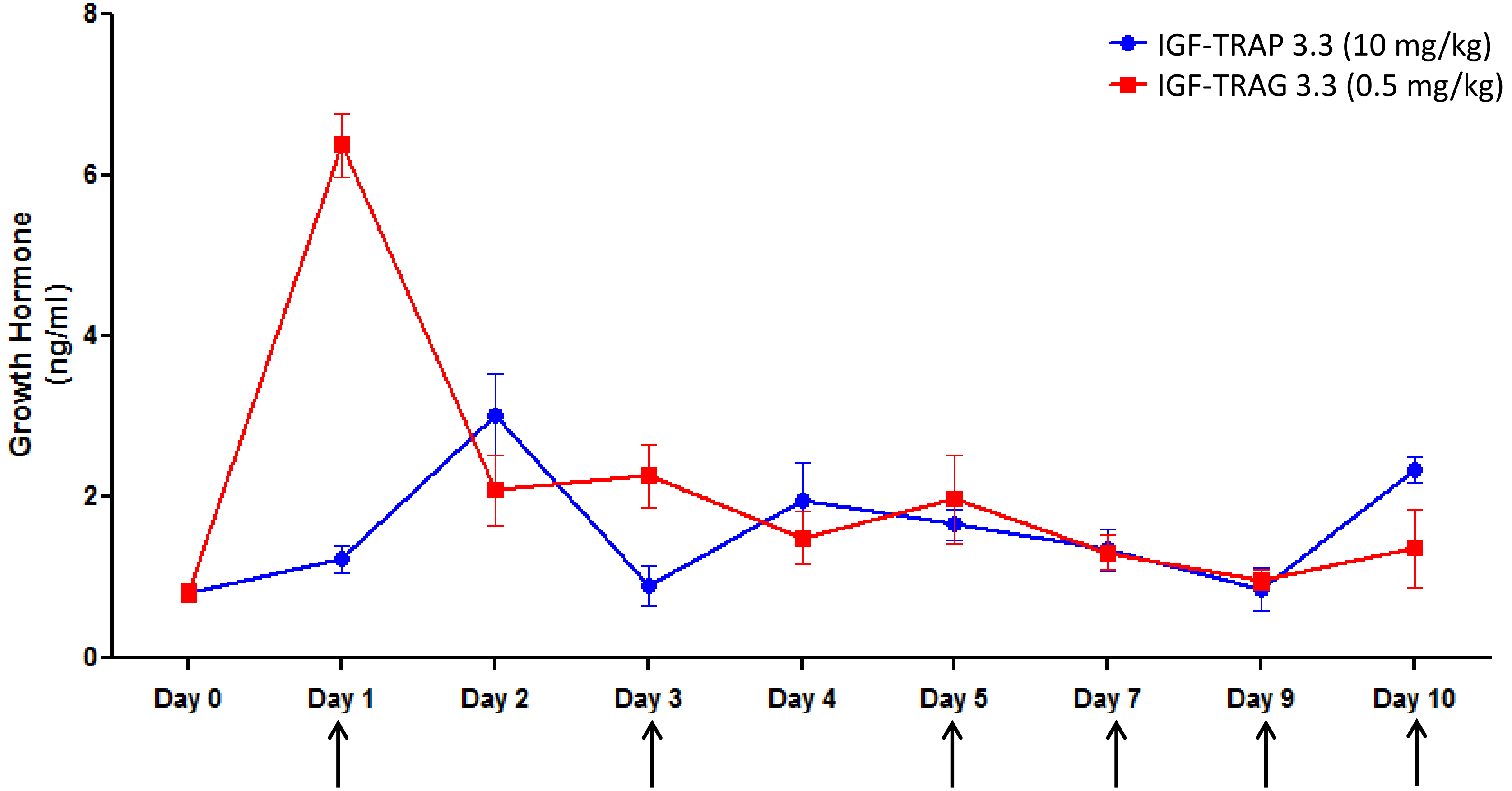

Supplementary Fig S3
